# Endemic status of urogenital schistosomiasis and the efficacy of a single-dose praziquantel treatment in unmapped rural farming communities in Oyo East Local Government Area, Oyo State, Nigeria

**DOI:** 10.1371/journal.pntd.0012101

**Published:** 2024-04-15

**Authors:** Alexander B. Odaibo, Adenike K. Komolafe, Taiwo O. Olajumoke, Kanyinsola D. Diyan, Damilare A. Aluko, Oluwatunmininu A. Alagbe, Oluwafemi A. Ajagbe, David B. Olarinloye

**Affiliations:** 1 Parasitology Research Unit, Department of Zoology, University of Ibadan, Ibadan, Nigeria; 2 Adeyemi Primary Health Care Center, OyoEast Local Government Health Authority, Oyo, Nigeria; 3 Oyo State Primary Health Care Board, Ibadan, Oyo State, Nigeria; Natural History Museum, UNITED KINGDOM

## Abstract

**Background:**

Schistosomiasis is endemic in Nigeria, and the treatment is largely concentrated on children enrolled in schools. Consequently, the coverage of non-enrolled school-aged children is often neglected. Ajagba and Awosan are two communities in Nigeria that have never had any control intervention. Hence, this survey was designed to determine the endemicity of urogenital schistosomiasis and to evaluate the efficacy of a single-dose praziquantel in the communities.

**Methods:**

Urine sample (10 mL) of each participant from Ajagba and Awosan communities was filtered through 12μm polycarbonate filter. The filter was placed on a microscope slide, and stained with a drop of 1% Lugol iodine solution. The stained slides were examined under the microscope and the numbers of *S*. *haematobium* eggs were counted. Water contact sites were searched for snail hosts and the snails collected were shed for *Schistosoma* cercariae. Data were analyzed using SPSS version 24.0 and the significance level was set at 95%.

**Results:**

The overall prevalence of infection in the Ajagba community was 45.6% with a mean intensity of 61.1 ± 144.5 eggs/10 mL of urine, while the prevalence of infection in the Awosan community was 5.7% with a mean intensity of 1.4 ± 6.8 eggs/10 mL of urine. The school-aged children had a prevalence and mean intensity of infection of 73.1% and 111.6 ± 177.9 eggs/10 mL of urine, respectively. Following treatment, women had a higher egg reduction rate than men (p = 0.0283). *Bulinus globosus* were found in Ajagba but not in Awosan, with 5.7% shedding *Schistosoma* spp, cercariae.

**Conclusion:**

Urogenital schistosomiasis was hyperendemic in the Ajagba community, and hypoendemic in the Awosan community. The presence of *Bulinus globosus* supported the transmission of the schistosomiasis in the Ajagba community. Communities where schistosomiasis is still actively transmitted in Nigeria should be identified for effective intervention through the MDA programs.

## Author summary

*Schistosoma haematobium* causes urogenital schistosomiasis that is endemic and widely distributed in Nigeria. For effective control strategies, it is important to continue to identify and classify communities where the disease is still been transmitted. A cross-sectional community-based survey was conducted in two closely located rural farming communities that had not had any control interventions, to determine the status of the disease in the communities, and initiate the administration of medicine. Urine samples were collected from volunteering participants and examined for *S*. *haematobium* eggs. Of 167 participants who submitted urine samples in the two communities, 32.9% were infected. The Ajagba community had more infected participants (45.6%) than the Awosan community (5.7%). All age groups were infected in the Ajagba community, but school-aged children were more infected than any other age group. Snail intermediate hosts were found at the water contact site in Ajagba but not at water contact site in Awosan. The study suggests that communities with natural freshwater bodies that harbor appropriate snail intermediate hosts are at greater risk of infection.

## Introduction

Schistosomiasis is a neglected tropical disease that is endemic in every region of Nigeria, and is affecting all age groups, with varying prevalence and intensities of infection. It is estimated that about 1 billion people globally are at risk of infection, with about 251.4 million people requiring preventive treatment in 2021 [1]. Sub-Saharan Africa accounts for 93% (192 million cases) of the world estimate of 207 million cases of schistosomiasis, and Nigeria has the highest prevalence of infection (29 million cases) [2].

There are two major forms of schistosomiasis, urogenital schistosomiasis caused by *Schistosoma haematobium*, and intestinal schistosomiasis, caused by *S*. *mansoni*, that are commonly reported in Nigeria. Urogenital schistosomiasis is most widely reported in Nigeria, affecting individuals living in most rural, underprivileged urban or peri-urban settings with limited access to clean water, inadequate sanitation, and hygiene services [3–5]. The disease is also commonly found in fishing and agricultural dominant communities where direct interactions with water increase the risk of contracting the disease [6,7]. Generally, schistosomiasis transmission is highly associated with the presence of appropriate freshwater snail intermediate hosts, in which the larval stages of the schistosome parasite develop; and human contact with the water inhabited by the infected snails results in infection [8]. Some species of the bulinids (*Bulinus globosus* and *B*. *truncatus)* act as intermediate hosts of *S*. *haematobium* in Nigeria. *Bulinus globosus* is widely distributed and common while *B truncatus* is restricted in distribution, but there are instances where both species are sympatric in distribution [9], with great implications for the transmission of urogenital schistosomiasis. The strategy for the control of schistosomiasis in Nigeria emphasizes mass drug administration (MDA) of praziquantel (PZQ) through schools, and the country accounts for an estimated 24.5% of the global population that requires preventive chemotherapy for schistosomiasis [7]. The impact of annual treatments on the prevalence and intensity of schistosome infection in various control programs has led to a reduction in high-intensity infections [8]. Endemic communities in Nigeria and other African countries, such as Cameroon, Ethiopia, Democratic Republic of the Congo, that have had intervention through MDA of praziquantel in schools have experienced significant reduction in prevalence and intensities of *Schistosoma haematobium* infections [10–16]. It was estimated that Nigeria had a 68.7% overall relative prevalence reduction for *S*. *haematobium* infection in 2015–2019 [17], following years of MDA with praziquantel. The major limitation of this strategy is that MDA does not prevent re-infection, and in most cases, high-risk groups, like out-of-school and preschool-aged children, and adults that engage in high-risk professions are excluded [18]. Consequently, small rural communities, very often with high-risk groups, without schools will be excluded from mapping, the visual representation of the geographical distribution of schistosomiasis in Nigeria and would not have access to preventive chemotherapy and are consequently excluded from intervention programs, particularly when no special provisions are made for their inclusion. Hence, the untreated infected individuals in these communities experience severe adverse health consequences associated with untreated chronic schistosomiasis [19]. It is therefore important to continue to identify and classify communities where schistosomiasis is still been transmitted so as to provide empirical data for effective control interventions in such endemic communities.

There are two farming communities, Ajagba and Awosan, located in Oyo East Local Government Area of Oyo State, Nigeria, that have never had any control intervention, and there was a recent report of some of the children passing blood in their urine (gross hematuria) (Oladoyinbo, personal communication). Hence, a cross-sectional, community-based study was designed to provide baseline information on the endemic status of urogenital schistosomiasis and evaluate the efficacy of praziquantel treatment in the two rural farming communities that had not benefitted from MDA programs, in Oyo East Local Government Area, Oyo State, Nigeria.

## Materials and methods

### Study area

**Fig 1:**
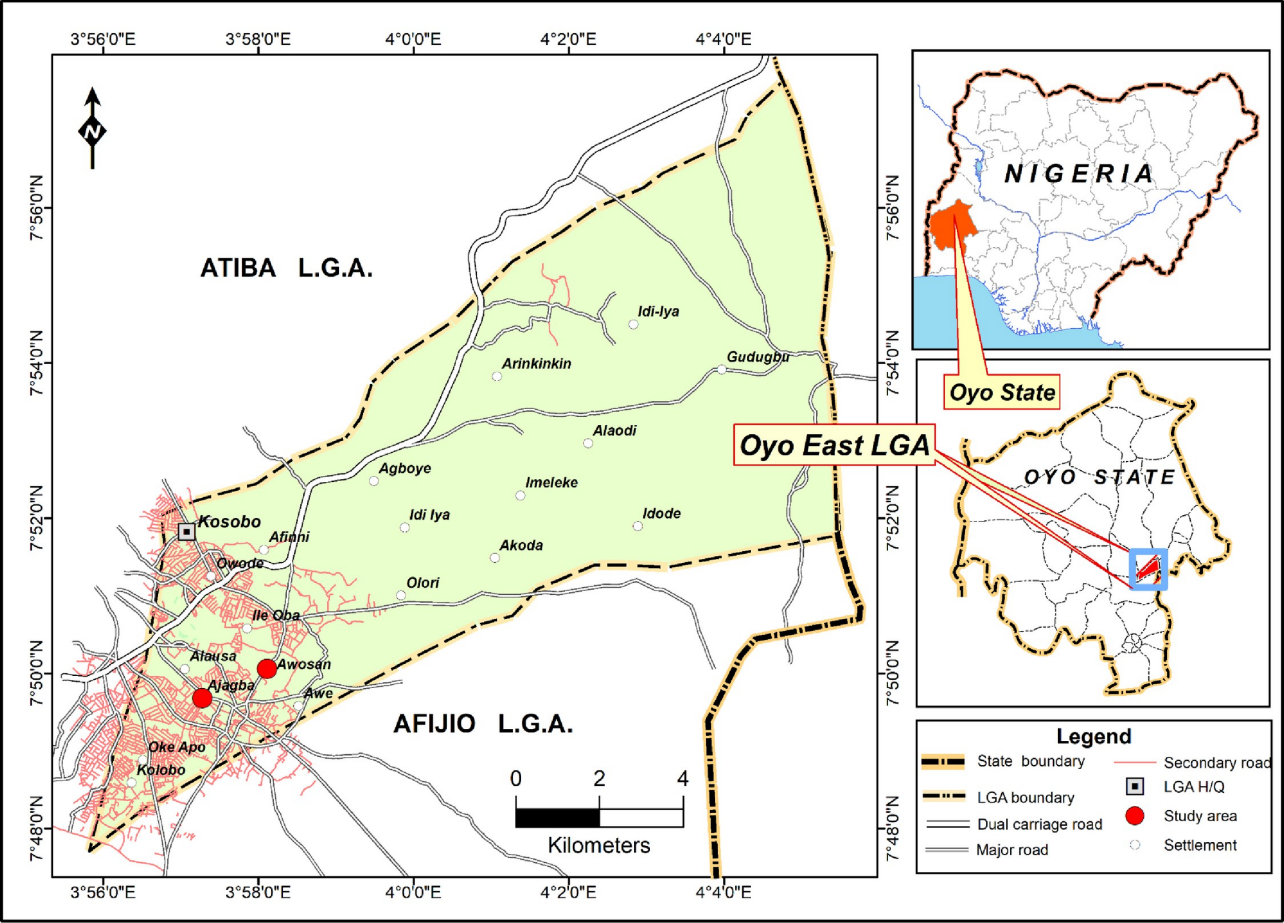
Map showing the Ajagba and the Awosan communities in Oyo East Local Government Area. **The map was created in the ArcGIS software program, and the source of the basemap shapefile was the U.S. Geological Survey (USGS). Base layer of the map**: https://www.openstreetmap.org/#map=6/9.117/8.674.

Oyo East Local Government area (Fig 1) is one of the 33 local government areas (LGAs) of Oyo State, with its headquarters at Kosobo. It has a land area of about 144 km^**2**^ and a population of 123,846. It lies between the Latitude 7.68^**o**^ N– 7.87^**o**^ N and Longitude 3.97^**o**^ E– 4.0213^**o**^ E. The study was conducted in the Ajagba ward, one of the ten wards in the LGA. The ward has two distinct seasons, the wet and the dry seasons. The wet season is between the months of March and April, and it lasts till October, while the dry season spans from November to March. The mean annual rainfall is about 1,600 mm with its peak in July [20]. The vegetation of the area consists of the Guinea Savanna zone. Two farming communities, Ajagba and Awosan villages, adjacent to each other, were surveyed for urogenital schistosomiasis. There were 30 households in Ajagba and 24 in Awosan, with about 950 inhabitants in the two communities. They lacked potable water supply; hence they depended on the River Iwodo, which drains the area and serves as a water source for domestic needs, recreational purposes, and farming activities. The river flows all year round and the two communities utilize different water contact sites along the path of the river. Awosan had only one water contact site, which was located about 400 m from the community, while Ajagba had two water contact sites; the first, “Odo Pako” was located about 300 m from the community, and was regularly used for washing clothes, bathing, fishing, and swimming, while the second, “Odo Apata”, located about 500 m from the community was solely used for domestic purposes. During the wet season, the inhabitants of the Ajagba community also frequent “Odo Pako” for fishing and collection of aquatic snails, *Lanistes libycus* and *Pila ovata* for consumption. Odo Pako has bamboo trees (*Bambusa* sp.) and other plants along the shores. The bamboo trees provided shade, and the fallen leaves and branches provided substrates for the attachment of aquatic snails. The water contact site at the Awosan community had no trees but a few vegetation along the shores.

There were no schools and no sanitary facilities in the two communities. There was a primary health care center located in the Ajagba community that lacked adequate equipment and facilities for effective health care services, which also served the various other communities in the Ajagba ward. The two communities, Ajagba and Awosan are located approximately 12–14 km from Oyo town, in Oyo LGA and were 1 km apart from each other.

### Ethics statement

The study protocol was reviewed and approved by the Ethical Committee of Oyo State Ministry of Health, (AD 13/474/44642^A^). Local permission for the study was obtained from the Medical Officer of the primary health care center at Oyo town, and the village head (“Bale”). The study aims and procedures were fully explained in the local language (Yoruba) to all participants prior to the commencement of the study. All participants gave written consent or assent to be registered in the study, following the health talks, which include information sharing on how human beings become infected with schistosome parasites, the signs and symptoms of chronic schistosomiasis, the risk factors of schistosomiasis and the treatment. In addition, written consent was obtained from the parents of the children registered in the study. All participants that were positive for *S*. *haematobium* infection were treated with praziquantel (40 mg/kg of body weight).

### Inclusion and exclusion criteria

Everybody who was a permanent resident of any of the two communities, who volunteered, was eligible to participate in the study. Visitors to the communities, those that declined participation, preschool–aged children that could not produce urine at the time of sample collection, and those who collected sample bottles but did not submit urine samples were excluded from the study.

### Study design

The cross-sectional, community-based survey was conducted between April and May 2022. The study was designed to determine the endemic status of urogenital schistosomiasis and the efficacy of a single-dose praziquantel treatment in two farming communities. Before the commencement of the study, the aim and objectives of the study were explained to the head of each community and the heads of the households in the communities. During the study, the recruitment of participants was done through house-to-house visits, with the assistance of health officials from the primary health care center in the Ajagba community.

### Data collection

Residents of the Ajagba community, who voluntarily agreed to participate, were gathered at the primary health care center located in the community, while residents of the Awosan community, without a primary health care center, were gathered at a suitable place for more briefing and sample collection. Health talks and all the information concerning the study were given in the local language (Yoruba). Before sample collection, participants provided answers to the questionnaire (S1 Questionnaire). The information about individual’s socio-demographic characteristics, water-contact practices, were collected. The questionnaire was filled out by qualified research staff, based on the information provided by the participants. Thereafter, all the participants received sample bottles and they were instructed to go and bring their urine samples. The urine samples were collected between 10 am and 2 pm into 50 mL clean containers with wide mouth and screw caps. Samples were then transported in cooling boxes to the Parasitology Research Laboratory, Department of Zoology, University of Ibadan, Ibadan, Nigeria for examination.

### Sample size

To estimate the sample size for the survey, the previously reported prevalence (17.4%) of urogenital schistosomiasis in the region [21] was used. The sample size was calculated using the formula of Lwanga et al. [22],

$$n=\frac{Z^{2}[P(1-P)]}{d^{2}}$$

where Z is the Z-value for the 95% confidence interval, that is alpha = 5% [Z = 1.96]

P is the expected proportion of the outcome to be infected (p = 0.17), q = 1- p = 0.826, d is the precision for the given confidence interval expected as a decimal (d = 0.06). The minimum estimated sample size for the two communities was 153. However, considering the small population sizes of the communities, all residents who volunteered to participate in the study were enrolled.

### Urine examination

Each sample was examined visually for gross hematuria and tested for microhematuria, proteinuria and leukocyturia using reagent strips (Combur-9 test, manufactured by Analyticon Biotechnologies, Germany). The reagent strip changes color when immersed in, and then removed from the urine sample. It is semi-quantitative, as it provides a positive or negative reaction for a parameter and also provides an estimation of a quantitative result (as trace,1+,2+,3+ and 4+) for the same parameter. Thereafter, the urine sample was agitated to ensure a random distribution of eggs, and 10 mL of each sample was filtered through a 13 mm diameter, 12μm polycarbonate filter (Sterlitech Corporation, Kent WA, USA). The filter, which was held in a plastic 13 mm holder was carefully removed and placed on a clean microscope slide and stained with a drop of 1% Lugol iodine solution. The stained slides were examined under the binocular compound microscope and the number of terminally spined *S*. *haematobium* eggs was counted and recorded. Only a single slide and single urine sample were used for the diagnosis of *S*. *haematobium* infection in this study. Following the World Health Organization (WHO) standard, the categories of intensities of infection with *S*. *haematobium* was classified as light (< 50 eggs/10 ml of urine) or heavy (≥ 50 eggs/ 10 ml of urine) [23].

### Treatment of infected participants and monitoring of adverse events

All infected participants were treated with 40 mg/kg of PZQ. A total of 52 infected participants were treated with PZQ, and only those that were available for the follow–up study, were recruited. Those that were not available, were those who would have left their houses early in the morning for their farming activities.

### Follow-up survey

A follow-up survey of infected participants treated with 40 mg/kg body weight of praziquantel at the Ajagba community was conducted 2 weeks (14 days) following the treatment. Urine samples were examined according to the procedure conducted in the baseline survey. The number of *S*. *haematobium* eggs was counted and recorded. Egg reduction rates were determined to evaluate the efficacy of praziquantel in the farming community.

### Snail survey

The two communities had separate water contact sites along the Iwodo River that drains the area. All water contact sites were searched for freshwater snails using long hand scoops and hand-picking for 15 minutes at each water contact site. Snails collected were transported to the Parasitology Laboratory of the Department of Zoology, University of Ibadan, Ibadan, for sorting into species types for identification. Snails were identified using the morphological identification keys for West African freshwater snails by Brown and Kristensen [24].

### Examination of snails for patent infection

The pulmonate snails, *Bulinus globosus* collected from the water-contact sites were examined for patent infections i.e., snails that are shedding cercariae [25,26], by putting individual snails into a shedding vial containing 4–5 mL of dechlorinated tap water and then exposed to sunlight for about one hour. The vials were observed under the microscope for cercariae. Snails shedding furcocercous cercariae were considered infected. The shed cercariae were identified using the morphological identification guide by Frandsen and Christensen [27].

### Statistical analysis

The data were analyzed using SPSS version 24.0 (SPSS, Inc., USA). Proportions of *S*. *haematobium* infections were compared between different groups, such as age and sex, using Pearson Chi-square test [χ^2^]. Wilcoxon signed-rank test was used to compare differences in the mean intensity of infection at baseline and post treatment. The Mann-Whitney U test was used to compare differences in the mean intensity between sex and community. The schistosome egg reduction rate (ERR %) following treatment with the single-dose 40 mg/kg body weight with praziquantel was used to assess the efficacy of praziquantel treatment. It was calculated as [1 –(Arithmetic mean egg counts per 10 mL of urine at follow up / Arithmetic mean egg counts per 10 mL of urine at baseline treatment)] x 100, and the reference drug efficacy (≥ 90%) of praziquantel (600 mg tablet) for *S*. *haematobium* infection [7,23] was adopted in this study. The arithmetic mean egg count values of all participants (infected or not) were used to assess egg counts in this study. The Confidence intervals [CI] were calculated using a confidence interval calculator. A p-value of < 0.05 was taken to indicate statistical significance.

## Results

### Characteristics of the study population

A total number of 173 volunteered participants were recruited into the study, but only 167 (96.5%, 95% CL = 93.8–99.2%) submitted their urine samples for examination, and 114 (68.3%, 95% CL = 59.9–76.8%) of them were from the Ajagba community, while 53 (31.7%, 95% CL = 23.2–40.2%) were from the Awosan community. The overall mean age of the participants who provided urine samples was 23.4 ± 20.5 years (range: 2–74 years), and 70 (42.0%, 95% CL = 32.7–47.5%) were males, while 97 (58.0%, 95% CL = 52.5–67.3%) were females. The preschool-aged children (2–4 years) constituted 9.6% (95% CL = 5.1–14.1%) of the study participants, while the school-aged children (SAC) (5–15 years) constituted 47.0% (95% CL = 39–55%) of the participants (Table 1).

**Table 1:** General characteristics of communities and participants in the urogenital schistosomiasis survey in the Ajagba and the Awosan communities.

Characteristic	Number	Percentage	95% Confidence limits
Sex			
Male	70	42	32.7–47.5
Female	97	58	52.5–67.3
Age (yrs.)			
2–4	16	9.6	5.1–14.1
5–15	78	46.7	39–55
16–85	73	43.7	32.3–55
Community			
Ajagba	114	68.3	59.9–76.8
Awosan	53	31.7	23.2–40.2
Distance from Stream (meters)			
Ajagba	>200	-	-
Awosan	>200	-	-
Primary health facility			
Ajagba	1	-	-
Awosan	0	-	-

### Overall prevalence and arithmetic mean intensity of infection by community

There was a significant difference (χ^2^ = 26.1475; p = 0.00001) in the overall prevalence of urogenital schistosomiasis between the Ajagba and the Awosan communities at the baseline. The overall prevalence of infection in the Ajagba community was 45.6% (37–55%) with an overall arithmetic mean intensity of infection of 61.1 ± 144.5 eggs/10 mL of urine, while the overall prevalence of infection in the Awosan community was 5.7% (1–12%) and the overall arithmetic mean intensity was 1.4 ± 6.8 eggs/10 mL of urine (Table 2). The morbidities associated with *S*. *haematobium* infection, microhematuria (51.8%, 95% CI = 43–61%), proteinuria (62.5%, 95% CI = 54–71%), and leukocyturia (54.4%, 95% CI = 45–64%) in the Ajagba community were higher than those in the Awosan community, with microhematuria (11.5%, 95% CI = 3.0–20%), proteinuria (47.2%, 95% CI = 34–61%), and leukocyturia (9.4%, 95% CI = 2.0–17%) (Table 2).

**Table 2:** The prevalence and the arithmetic mean intensity of *Schistosoma haematobium* infections in the Ajagba and the Awosan communities by sex.

Community surveyed	Sex	No. examined	No. infected	Prevalence (%), [95% CI]	P-value	Arithmetic mean ($\bar{X}$ ± SD) Eggs/10 mL Of urine, [95% CI]	P-value	Micro- hematuria (%), [95% CI]	Proteinuria (%), [95% CI]	Leukocy- turia (%), [95% CI]
Ajagba	Male	48	22	45.8 [37–55]	χ^2^ = 0.0016; p = 0.96802	57.1±139.6 [17.6–96.6]	0.4902	53.3 [39–67]	66.6 [53–80]	41.7 [28–50]
	Female	66	30	45.5 [37–55]		64.1±149.1 [28.1–100.0]		50.7 [39–63]	59.7 [48–72]	63.6 [52–75]
	Sub-total	114	52	45.6 [37–55]	χ^2^ = 0.0876; p = 0.76730	61.1±144.5 [34.6–87.6]	0.72634	51.8 [43–61]	62.5 [54–71]	54.4 [45–64]
Awosan	Male	22	1	4.5 [1.0–10]		1.3±6.0 [1.2–3.8]		13.6 [1–28]	45.5 [25–66]	0
	Female	31	2	6.5 [1.0–13]		1.5±7.4 [1.1–4.1]		9.7 [1.0–20]	48.4 [31–66]	16.0 [3.0–29]
	Sub-total	53	3	5.7 [1.0–12]		1.4±6.8 [1.0–3.2]		11.5 [3.0–20]	47.2 [34–61]	9.4 [2.0–17]
Grand total		167	55	32.9 [26–40]		41.7±122.6 [23.1–60.3]		38.3 [31–46]	56.9 [49–65]	40.1 [33–48]

* P-value for Chi squared test (χ2); significant at p < 0.05

** P-value for Mann-Whitney U Test; significant at p < 0.05

### Prevalence and arithmetic mean intensity of infection by age

Infections were detected among all age groups in the Ajagba community. The prevalence of infection among the preschool-aged children (2–4 years) in the Ajagba community was 18.2% (1.0–41%), with an arithmetic mean intensity of 13.1 ± 30.4 eggs/10 mL of urine, and all of them were heavily (> 50 eggs/10 mL urine) infected (Table 3). The school-aged children had a prevalence of infection of 73.0% (61–85%), with an arithmetic mean intensity of infection of 111.6 ± 177.9 eggs/10 mL of urine). Approximately 52.6% (37–68%) of them were heavily infected (≥50 eggs/10 mL urine), while 47.4% (32–63%) of them had light infection (1–49 eggs/10 mL of urine) (Table 3). In the Ajagba community, the age group 40–80 years had the least prevalence of infection (30.4%, 95% CI = 12–49%) and mean intensity of infection (1.1 ± 2.7 eggs/10 mL of urine). While in the Awosan community, no preschool-aged nor school-aged children were infected with *S*. *haematobium*. The prevalence of infection among the only age group (16–39 years) in the Awosan community that had infection was 21.4% (95% CI = 1.0–62%), with an arithmetic mean intensity of infection of 5.4 ± 12.7 eggs/10 mL of urine (Table 3).

**Table 3:** The prevalence and the arithmetic mean intensity of *Schistosoma haematobium* infection in the Ajagba and the Awosan communities in the Oyo East Local Government Area, by age.

Community surveyed	Age group (yrs.)	No. examined (n)	No. infected (n)	Prevalence (%) [95% CI]	Arithmetic mean intensity ($\bar{X}$±SD) Eggs/10 mL of urine, [95% CI]	Light intensity (1–49 Eggs/1o mL of urine) % [95% CI]	Heavy intensity (≥50 Eggs/ 10 mL of urine) % [95% CI]
Ajagba	2–4	11	2	18.2 [1.0–14]	13.1±30.4 [1.0–31.1]	0	100
	5–15	52	38	73.1 [61–85]	111.6±177.9 [63.2–160.0]	47.4 [32.0–63.0]	52.6 [37.0–68.0]
	16–39	19	5	26.3 [6.0–56]	15.2±46.2 [1.0–36.0]	60.0 [17.0–103.0]	40.0 [1.0–83.0]
	40–80	32	7	30.4 [12–49]	1.1±2.7 [1.0–2.0]	100	0
	Sub-total	114	52	45.6 [55–36]	61.1±144.5 [34.6–87.6]	53.8 [40.0 67.0]	46.2 [33.0–60.0]
Awosan	2–4	5	0	0	0	0	0
	5–15	26	0	0	0	0	0
	16–39	14	3	21.4 [1.0–62]	5.4±12.7 [1.0–12.1]	100	0
	40–70	8	0	0	0	0	0
	Sub- total	53	3	5.7 [1.0–12]	1.4±6.8 [1.0–3.2]	100	0
Grand total		167	55	32.9 [26–40]	41.7±122.6 [23.1–60.3]	56.4 [43.0–70.0]	43.6 [33.0–59.0]

### Prevalence and arithmetic mean intensity of infection by sex

There was no significant difference (χ^2^ = 0.0016; p = 0.968021) in the prevalence of infection between male (45.8%, 95% CI = 37–55%) and female (45.5%, 95% CI = 37–55%) in the Ajagba community at baseline. There was also no significant difference (p = 0.4902) in the arithmetic mean intensity of infection between male (57.1 ± 139.6 eggs/ 10 mL of urine) and female (64.1 ± 149.1 eggs/ 10 mL of urine) in the Ajagba community. A similar pattern of infection was observed in the Awosan community; the male had a prevalence of 4.5% (95% CI = 1.0–10%) while the female had a prevalence of 6.5% (95% CI = 1.0–13%) (χ^2^ = 0.0876; p = 0.76730). The arithmetic mean intensity of *S*. *haematobium* infection in males (1.3 ± 6 eggs/10 mL of urine) was not significantly different from the arithmetic mean intensity of infection in females (1.5 ± 7.4 eggs/ 10 mL of urine) (p = 0.72634) (Table 2).

### Treatment of infected participants

All the infected participants that presented themselves for treatment were given a single-dose of praziquantel at 40 mg/kg body weight. Minor adverse events were limited to vomiting (2), particularly among the preschool-aged children, abdominal pain (1), and dizziness (1). All reports were transient, and only lasted for few minutes.

### Follow-up survey after treatment in Ajagba community

**Fig 2:**
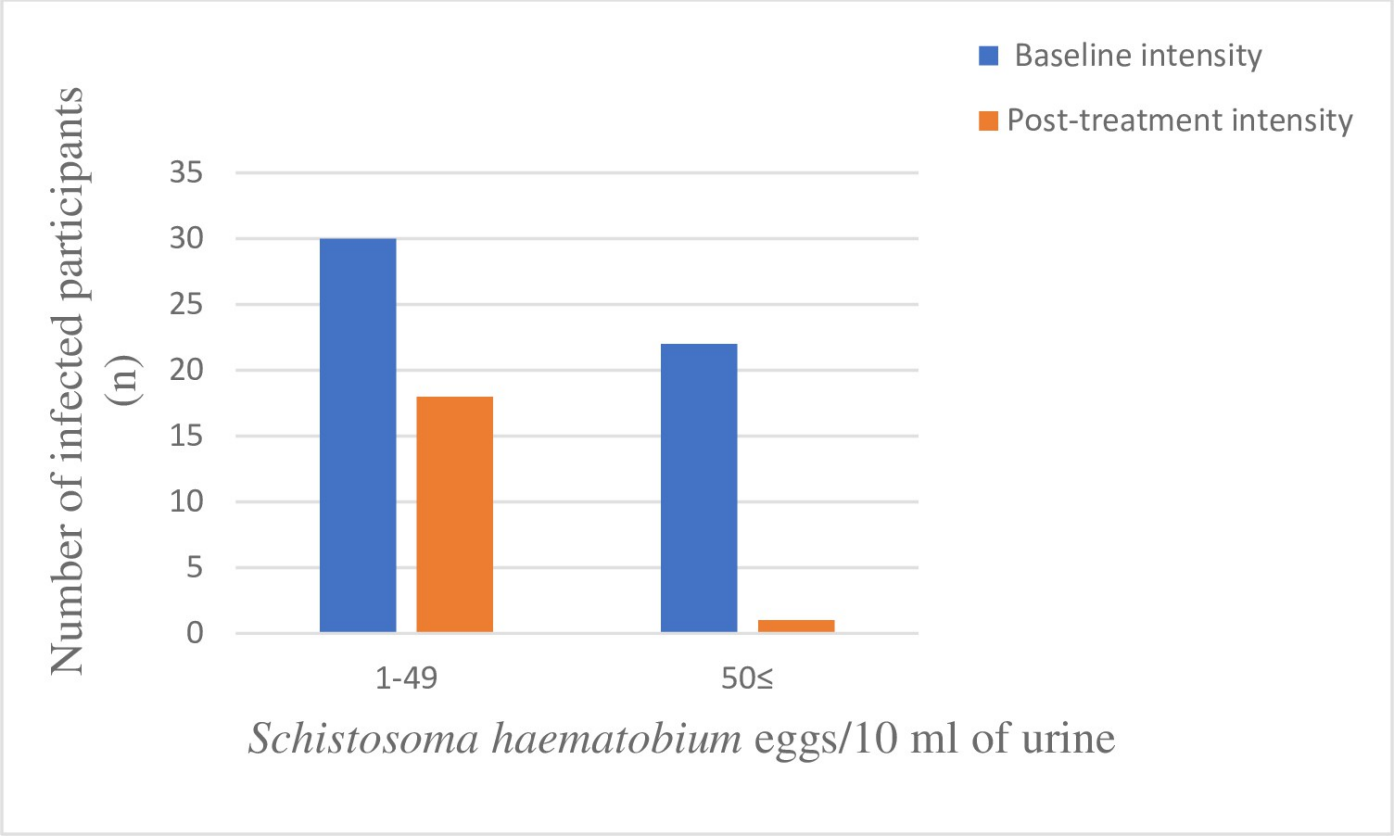
The categories of intensities at the baseline and the post-treatment surveys in the Ajagba community.

**Fig 3:**
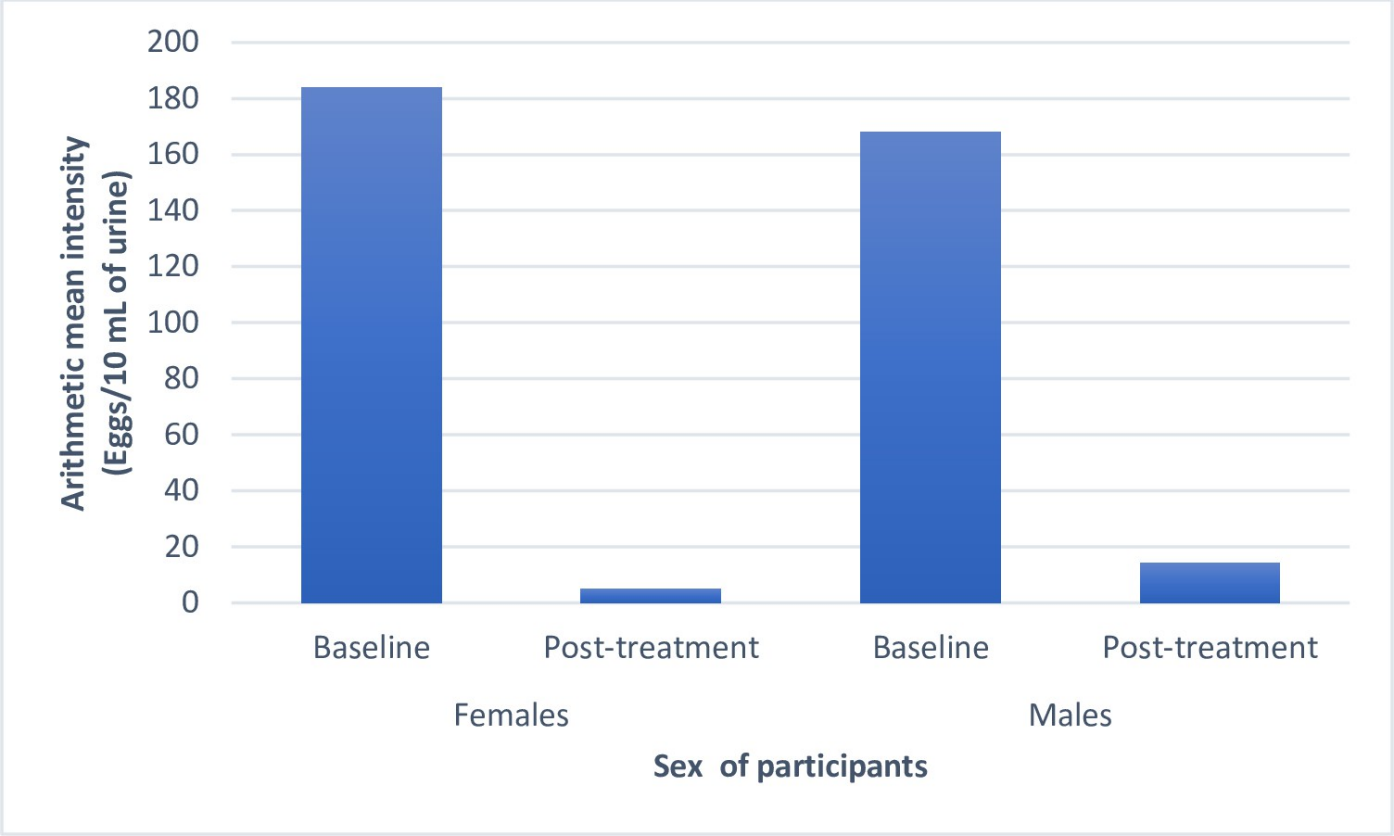
The mean intensities of infection at the baseline and the post-treatment surveys in the Ajagba community by sex.

The follow-up survey was carried out only in the Ajagba community with high endemicity of urogenital schistosomiasis. At the follow–up study 56% (95% CI = 45–60%) of the infected participants in the Ajagba community that participated were heavily infected (≥50 eggs/10mL) at baseline, however, week two (14 days) after treatment with praziquantel (40 mg/body weight), only few treated participants, who had heavy infection at baseline still had heavy infection after treatment, the majority of the infections persisting after treatment were in the light-intensity category (Fig 2). There was a significant reduction (χ^2^ = 64.381; p = 0.00001) in the proportion of infected participants with heavy infections. The egg reduction rate among participants that had light intensity (1–49 eggs/10mL) at baseline was 29.2% (95% CI = 4.0–55%) while the egg reduction rate among the heavily infected participants was 96.5% (95% CI = 87–105%) (S1 Table). There was a significant (p<0.05) difference in the egg reduction rates between baseline heavily and lightly infected participants (χ^2^ = 24.4905; p = 0.00001) in the Ajagba community. Overall, the egg reduction rate among the infected participants, following a single dose administration of praziquantel in the community, was 94.8% (95% CI = 86–103%). There was a significant difference (χ^2^ = 4.8082; p = 0.028325) in egg reduction rates by sex of participants in the Ajagba community. The female had a higher egg reduction rate of 97.2% (95% CI = 89–106%), than the male with a reduction rate of 91.6% (95% CI = 70–106%). The mean intensity of infection among the females, following the single-dose treatment was 5.1± 5.8 eggs/10 mL of urine, while the mean intensity of infection among the males was 14.1±21.8 eggs/10 mL of urine (Fig 3). The school-aged children with the highest prevalence and intensity of infection, had an egg reduction rate of 94.6% (95% CI = 88–100%), following treatment with praziquantel (40 mg/10 mL).

### Prevalence and the diagnostic values of microhematuria, proteinuria and leukocyturia in the Ajagba and the Awosan communities

**Fig 4:**
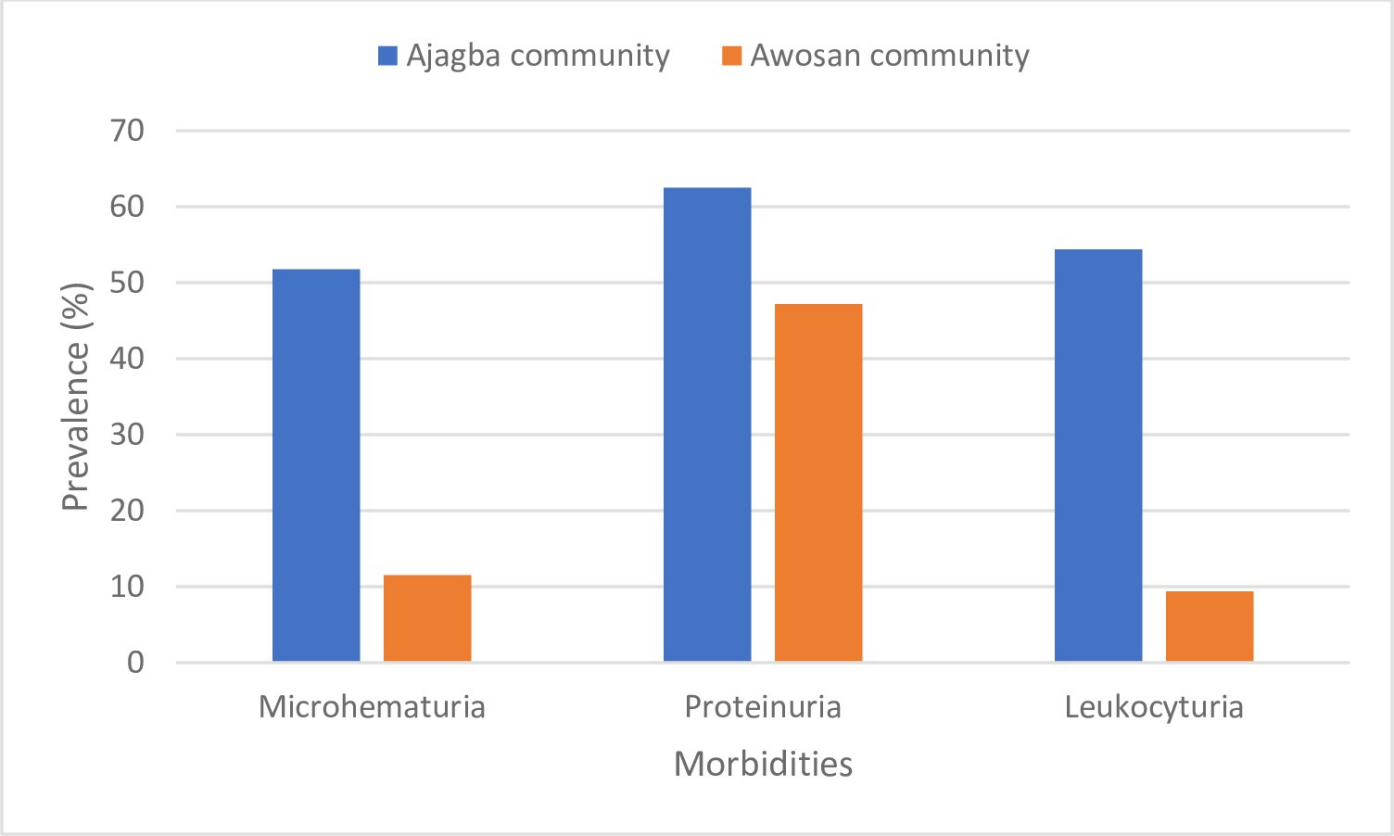
Prevalence of microhematuria, proteinuria and leukocyturia among participants at the Ajagba and the Awosan communities.

The morbidity associated with the infection in the two communities, such as microhematuria, proteinuria and leukocyturia were more prevalent in the Ajagba community than the Awosan community (Fig 4). The prevalence of proteinuria (47.2%, 95% CI = 34–61%) in the Awosan was high despite the low prevalence (5.7%, 95% CI = 0.5–12%) of urogenital schistosomiasis. Using microscopy as the ‘gold’ standard, the diagnostic values of microhematuria, proteinuria and leukocyturia, in the two communities were assessed. The results (S2 Table) showed that the three parameters microhematuria, proteinuria and leukocyturia had low false negative error rates (alpha error rates). The rates at which they failed to detect infected participants, were 21.1% (95% CI = 14–29%), 8.9% (95% CI = 4–14%) and 27.8% (95% CI = 20–36%) respectively, in the Ajagba community. While, on the other hand, microhematuria, proteinuria and leukocyturia had significantly high false negative error rates of 80.0% (95% CI = 69–91%), 90.0% (95% CI = 82–98%) and 87.5% (95% CI = 79–96%) respectively, in the Awosan community. Proteinuria had high (>50%) false positive error rates in the Ajagba and the Awosan communities. The positive predictive values of the three parameters, microhematuria (14.3%, 95% CI = 9.0–24%), proteinuria (2.0%, 95% CI = 1.0–6.0%), and leukocyturia (9.1%, 95% CI = 1.0–16%) were very low in the Awosan community, but the negative predictive values were high for the two communities. The diagnostic odds ratio (DOR) showed that microhematuria (23) had a better test performance than proteinuria (7.7) or leukocyturia (4.8) in the Ajagba community.

### Freshwater snail species in the water-contact sites of the two communities

A higher diversity of fresh-water snail species was found in Ajagba compared to Awosan (Table 4), however, the focus in this study was on the *B*. *globosus* as they are intermediate hosts. Of the 35 *B*. *globosus* collected in Ajagba, two (5.7%) were observed to shed *Schistosoma* spp. cercariae. (Table 4).

**Table 4:** The freshwater snail species found and collected from the water-contact sites in the Ajagba and the Awosan communities.

Snail species	Type of snail	Ajagba community		Awosan community	
		No. collected	No. with patent *Schistosoma* infection [%]	No. collected	No. with patent *Schistosoma* infection
*Bulinus globosus*	Pulmonate	35	2 [5.7]	0	0
*Melanoides tuberculata*	Prosobranch	20		10	-
*Gyraulus costulatus*	Pulmonate	50	-	0	-
*Potadoma moerchi*	Prosobranch	30	-	12	-
*Lanistes libycus*	Prosobranch	10	-	0	-
*Pila ovata*	Prosobranch	15	-	9	-
Total		150	2 [1.3]	31	-

## Discussion

This is the first survey in these two farming communities to provide epidemiological data necessary for effective MDA with praziquantel in the unmapped rural communities. Getting treatment to these neglected communities would not only help to control schistosomiasis generally, but also reduced chronic morbidity associated with the disease in the populations. This study further shows that there are unmapped rural communities in Nigeria still with active transmission of urogenital schistosomiasis, that require deliberate arrangement for their inclusion in the national intervention programs. Following the WHO classifications of endemicity [7,23], the Ajagba community with a prevalence of infection of (73%, 95% CI = 61–85%) among school-aged children (5–15 years), is hyper-endemic (≥ 50%) for urogenital schistosomiasis. The Awosan community, with overall prevalence of 5.7%, where none of the school-aged children examined were infected, and there were no appropriate snail intermediate hosts at the water-contact site, has low endemicity of urogenital schistosomiasis. There is the possibility that the few infections in the Awosan community were not autochthonous infections. A similar pattern has been observed in low-transmission setting in Tanzania, where there were no appropriate snail hosts or infections in snails at the water-contact sites, indicating that there was no local transmission, and the few cases of human infections were said to be imported cases [26]. This study supports the introduction of environmentally friendly snail control methods to complement MDA programs, as an opportunity for maintaining low-prevalence of schistosomiasis in endemic communities in Nigeria. The high endemicity observed in the Ajagba community showed that the community had not been reached with the preventive chemotherapy program, particularly as there were no schools in the community, and the current national MDA program in Nigeria is school-based. Generally, low prevalence of infection has been reported in areas that have benefitted from the MDA program in Nigeria [11,13,14], and some other African countries such as Ghana, Mali, and Senegal [16,17]. In this study, urogenital schistosomiasis was recorded in all age groups in the Ajagba community, including the preschool-aged children (2–4 years old) with heavy infections, who were probably passively exposed to infection by their mothers or caregivers [26], since the preschool-aged children would not have the capacity to actively engage in any water-contact activity, particularly in water contact sites that are located far away from the communities. This finding has further brought to fore the importance of urogenital schistosomiasis among this age group that is currently ignored in the MDA programs in Nigeria. The school-aged children had the highest prevalence and mean intensity of infection (73.1%, 95% CI = 61–85%; 111.6 ± 177.9 eggs/10 mL of urine respectively). This agrees with the general trend in Nigeria, in which prevalence and intensity of infection increase with age and peak in the school-aged groups, due to their high and long duration of exposure when fetching and playing in water [27]. There was no significant difference between infections in males and females, this was similar to the pattern reported [28,29] in some other communities in Nigeria. This may be because the water-associated behaviors were similar for the younger age groups that constituted the bulk of the statistical population in this study or because of high endemicity, the infection was widespread in the community, such that the difference between males and females was not obvious [30]. However, some other studies in different parts of Nigeria [3,14,21,31–36] and other endemic countries in Africa, such as Malawi [37], Sierra Leone [38] and Cameroon [19], have shown that the males always had higher prevalence and mean intensities of infection than the females, when the males dominated the activities with greater exposures to water. There are a number of other surveys in Nigeria that have also shown that the females were more infected than the males [39–41], when the water-related activities were greater for the females than the males in such endemic communities. The relationship between sex and risk of infection is therefore equivocal and varies with the cultural background of the people [19,27,30]. The relationship may vary from one region of the country to another and from one transmission season to another, even within the same endemic communities [42]. Whatever may be the case, it is often partly explained by differences in the social and occupational roles taken up by each participant that could affect water-related contact, including frequency, duration, and level of submergence, which may be influenced by age and cultural beliefs [30]. Therefore, any integrated strategy for the prevention and control of schistosomiasis must be predicated on the empirical data from each endemic community or region. The significant differences in prevalence and intensity of infection between the two communities, Ajagba and Awosan, that were only one kilometer apart, showed how focal *Schistosoma haematobium* transmission could be. The participants in the two communities engaged in water-contact activities in the same River Iwodo, but at different water-contact points. The water-contact point at the Ajagba community harbored more snail species, including *Bulinus globosus*, though with low prevalence of patent infection. Low prevalence of patent infection among snail species in Africa are common [43], particularly when only cercariae-shedding is used to determine infections among snail intermediate hosts. The water-contact point at Awosan had fewer snail species and no pulmonate snails that could transmit *Schistosoma haematobium* were collected during the study. This may partly explain why there was a high prevalence of infection in the Ajagba community and very low prevalence of infection in the Awosan community. Similar association between the presence of obligatory snail hosts and the prevalence of human infection has been observed in endemic communities in Kenya [44] and Ethiopia [45].

The single-dose treatment in the Ajagba community, with heavy infection, produced significant reduction in egg burden (p = 0.00008) among infected individuals at week two post-treatment. The reduction in egg burden among heavily infected participants (≥ 50 eggs/ 10mL of urine) was significantly higher (p = < 0.00001) than the egg reduction among those with light infection (1–49 eggs/10 mL of urine). Similar patterns of egg reduction had been reported in Cameroon [46] and Ethiopia [45]. The significant egg reduction among heavily infected individuals, in this study, would impact positively on the severe morbidity and disease transmission in the Ajagba community. Generally, in sub-Saharan Africa, praziquantel treatment has drastically reduced morbidity and transmission of schistosomiasis [17,47–49]. The single-dose of praziquantel at 40 mg/kg body weight, in this study, appeared to be more effective against heavy infections than the light infections. The lower reduction rates among lightly infected participants should be given the desired attention, as light infections, if untreated, can continue to cause considerable morbidity [15,50–52]. This may be prevented with repeated doses of PZQ, which can increase the efficacy of the treatment [53]. The overall egg reduction rate observed at week two, in this study, was above the 90% egg reduction rate threshold recommended for assessing the efficacy of anthelmintic drugs [23], indicating a satisfactory result. This study underscores the need to extend the use of preventive chemotherapy to support wider rural populations in Nigeria. Endemic communities, like the Ajagba community, where there were no schools, the available health facilities should be equipped to provide access to treatment with praziquantel to control morbidity due to schistosomiasis in all infected individuals. The predictive performance of each of the three reagent strip parameters in detecting infections with *Schistosoma haematobium* in the Awosan community, a low-prevalence setting, was very poor. This has great implications for the use of any of the parameters (microhematuria, proteinuria, and leukocyturia) for rapid diagnosis of urogenital schistosomiasis in low endemic areas or in monitoring the success of control measures in areas where prevalence has been reduced due to control programs. The low positive predictive values recorded in the Awosan community indicated that high proportions of truly uninfected participants, by microscopy, had positive test results with reagent strips. The negative predictive values, recorded in the Awosan community were however, very good. These findings corroborate the observations of Schulz and Grimes [54], that in low-prevalence settings, even excellent tests would have poor predictive positive values, while the negative predictive values would be nearly perfect. Diagnosis of urogenital schistosomiasis in low-prevalence settings, using the reagent strips, should be followed-up with microscopy in individuals who have positive tests results to determine if the disease is truly present [54].

In the Ajagba community, the DOR showed that microhematuria had a better test performance than proteinuria and leukocyturia in the Ajagba community. Similar findings had been reported for other endemic communities [55] in Nigeria.

### Limitations of study

The limitations of our study include, first, the moderate sample size used for the survey. The sample size is however, anticipated to be adequate, having used the recommended procedure [22] for a minimum sample size estimate for a population survey. Second, we did not do repeated urine filtration for microscopy. This would have increased the chances of finding more eggs of the parasite in the urine. Third, the use of only patent infections (cercariae shedding) to determine the prevalence of infection among snails was a limitation. If both patent and prepatent snail infections were determined, a more sensitive measure of infection among snails may have been observed. Additionally, molecular identification of the *Bulinus* and cercariae species found in Ajagba would have confirmed fresh water transmission sites for either human or animal schistosomiasis.

### Conclusion

Urogenital schistosomiasis was hyperendemic (≥ 50%) in the Ajagba community, and hypoendemic (<10%) in the Awosan community. All age groups were infected in the Ajagba community, but the preschool-aged and school-aged children in the Awosan community were not infected. The single-dose administration of praziquantel was very effective in the treatment of the disease in the Ajagba community. *Bulinus globosus*, a pulmonate freshwater snail hosts, was incriminated in the transmission of the disease in the Ajagba community. It is auspicious to continue to identify and classify communities where schistosomiasis is still been actively transmitted, for effective control intervention through the MDA programs in Nigeria.

## Supporting information

S1 Questionnaire Questionnaire for the survey of schistosomiasis in Oyo East Local Government, Oyo State, Nigeria. (DOCX)

S1 Table The egg reduction rates by sex and the categories of intensities of infection among the infected participants, at week two post-treatment with praziquantel (40 mg/kg body weight) in the Ajagba farming community. (DOCX)

S2 Table The diagnostic values of urinalysis with the reagent strips for urogenital schistosomiasis in the Ajagba and the Awosan farming communities in Oyo East Local Government Area, Oyo State, Nigeria. (DOCX)

S3 Table Urinogenital schistosomiasis project in Ajagba and Awosan communities in Oyo East Local Government Area field data. (DOCX)
