# Supplementary material for: Endemic status of urogenital schistosomiasis and the efficacy of a single-dose praziquantel treatment in unmapped rural farming communities in Oyo East Local Government Area, Oyo State, Nigeria
Source: PLoS Negl Trop Dis. 2024 Apr 15;18(4):e0012101. doi: 10.1371/journal.pntd.0012101 (PMC11045121; doi:10.1371/journal.pntd.0012101)
Supplement: S1 Questionnaire — (DOCX) [file pntd.0012101.s001.docx]

**Schistosomiasis in Oyo East Local Government, Oyo State, Nigeria.**

Good day Sir/ Ma, we wish to ask you some questions about your knowledge of urinary schistosomiasis, mode of prevention and your practices. You do not have to answer any question you are not comfortable with and you can end the interview at any time. Your response is voluntary. Any information you give to us will be confidential. Please, if you have any question to ask about this survey, feel free to do so.

Please note that your participation in this survey implies that you have understood and have freely given your consent to participate in this study. You can withdraw this consent at any time.

Consent ………………

LGA ………………… Settlement …..………………..

WARD………………. Geo-cordinates: Longitude…….Latitude……..

Section A: Socio-demographics Characteristics

1. Age in years (as at last birthday) …………….
2. Sex: 1. Male [ ] 2. Female [ ]
3. Marital Status: 1. Single [ ] 2. Married [ ] 3. Divorced [ ] 4. Separated [ ] 5. Others (Specify) ………………….
4. Ethnic group: 1. Yoruba [ ] 2. Igbo [ ] 3.Hausa [ ] 4.Others (specify) ……………..
5. Highest level of Education completed: 1. No formal Education [ ] 2. Primary [ ] 3. Junior Secondary [ ] 4. Senior Secondary [ ] 5.Tertiary [ ]
6. Occupation ………………1. Student [ ] 2. Farmer [ ] 3. Civil servant [ ] 4. Trader [ ] 5. Artisan [ ] 6. House keeper [ ] 7. Others (Specify)
7. Religion: 1. Christianity [ ] 2. Islam [ ] 3. Others (Specify) ………..

Section B: Knowledge of urogenital schistosomiasis.

1. Have you ever heard of urogenital schistosomiasis? 1. Yes [ ] 2. No [ ]
2. If yes, what is/are your source(s) of information? 1. Friends [ ] 2. Mass Media [ ] 3. Family [ ] 4. School [ ] 5. Hospital/ Health worker [ ] 6. Religious leaders [ ] 7. Others (specify) ………………….
3. How can people get urogenital schistosomiasis?

| People can get urogenital schistosomiasis by: | Yes | No | I don’t Know |
| --- | --- | --- | --- |
| Eating food contaminated by rat’s feaces and urine |  |  |  |
| Drinking infected water |  |  |  |
| Swimming in infected river or stream/spring |  |  |  |
| Entering infected river or stream/spring to fetch water |  |  |  |
| Entering infected river or stream/spring for fishing |  |  |  |
| Entering infected river or stream/spring to pass to another place |  |  |  |
| Entering infected river or stream/spring for bathing |  |  |  |
| Entering infected river or stream/spring to play |  |  |  |
| Others (Specify) |  |  |  |

Section C: Participants’ water contact practices.

1. Have you ever had contact with any river or stream/spring before? 1. Yes [ ] 2. No [ ]
2. If your answer is yes to Q11 is yes, for what reason? Choose all that apply.

| Reason for entering water | Yes | No |
| --- | --- | --- |
| Swimming |  |  |
| Fishing |  |  |
| Bathing |  |  |
| Fetching water |  |  |
| Snail collection |  |  |
| Wading through |  |  |
| Washing of cloth |  |  |
| Washing of plate and other cooking utensils |  |  |
| Playing |  |  |
| Others (Specify) |  |  |

1. Where do you get water for domestic use? Tick as appropriate.
2. Borehole
3. Well
4. Stream
5. River
6. Others (Specify)
7. Laboratory Result: Positive [ ] Negative [ ]

Thank you for your time.
