# Supplementary material for: Endemic status of urogenital schistosomiasis and the efficacy of a single-dose praziquantel treatment in unmapped rural farming communities in Oyo East Local Government Area, Oyo State, Nigeria
Source: PLoS Negl Trop Dis. 2024 Apr 15;18(4):e0012101. doi: 10.1371/journal.pntd.0012101 (PMC11045121; doi:10.1371/journal.pntd.0012101)
Supplement: S1 Table — (DOCX) [file pntd.0012101.s002.docx]

**S1 Table. The egg reduction rates by sex and the categories of intensities of infection among the infected participants, at week two post-treatment with praziquantel (40 mg/kg body weight) in the Ajagba farming community**

| **Category of intensity** | No. participants | Baseline  arithmetic mean intensity  (Ⴟ±SD)  Eggs/10 ml of urine, [95% CI] | Post-treatment arithmetic mean intensity  (Ⴟ±SD)  Eggs/10 ml of urine, {95% CI] | Wilcoxon Signed Rank Test (p-value) | Egg reduction rate (%) [95% CI] | Chi squared test (χ^2^);  p-value |
| --- | --- | --- | --- | --- | --- | --- |
| Light intensity  (1-49 eggs/10 ml of urine) | 12 | 9.6±8.3  [4.9 – 14.3] | 6.8±11.8  [1.0 – 13.5] | 0.25428 | 29.2 [4 - 55] | 24.4905; 0.00001^**^ |
| Heavy intensity  (≥50eggs/10 ml of urine) | 15 | 311±191.3  [214.2 - 407.8] | 10.9±17.7  [1.9 – 19.9] | 0.00064^**^ | 96.5 [87– 105] |  |
| Total | 27 | 177.0±207.5  [98.7 – 255.3] | 9.1±15.2  [3.4 – 14.8] | 0.00008^**^ | 94.8 [86– 103] | - |
| **Sex of participant** |  |  |  |  |  |  |
| Male | 12 | 168.5±215.1  [46.8 – 290.2] | 14.1±21.3  [2.0 – 26.2] | 0.0278^**^ | 91.6 [70– 106] | 4.8082  0.028325^**^ |
| Female | 15 | 183.9±208.5  [78.4 - 289.4] | 5.1±5.8  [2.2 – 8.0] | 0.0009** | 97.2 [89 – 106] |  |
| Total | 27 | 177.0±207.5  [98.7 – 255.3] | 9.1±15.2  [3.4 – 14.8] | 0.00008^**^ | 94.8 [86 – 103] |  |

^**^Significant at p<0.05
