# Supplementary material for: Endemic status of urogenital schistosomiasis and the efficacy of a single-dose praziquantel treatment in unmapped rural farming communities in Oyo East Local Government Area, Oyo State, Nigeria
Source: PLoS Negl Trop Dis. 2024 Apr 15;18(4):e0012101. doi: 10.1371/journal.pntd.0012101 (PMC11045121; doi:10.1371/journal.pntd.0012101)
Supplement: S2 Table — (DOCX) [file pntd.0012101.s003.docx]

**S2 Table. The diagnostic values of urinalysis with the reagent strips for urogenital schistosomiasis in the Ajagba and the Awosan farming communities in Oyo East Local Government Area, Oyo State, Nigeria**

| Community | Ajagba (n=114) | | | Awosan (n=53) | | |
| --- | --- | --- | --- | --- | --- | --- |
| Prevalence of UGS by egg microscopy (%) [95% CI] | 45.6 [37 - 55] | | | 5.7 [ 1– 12] | | |
| Dipstick Tests parameter | Microhematuria | Proteinuria | Leukocyturia | Microhematuria | Proteinuria | Leukocyturia |
| Prevalence (%) [95% CI] | 51.8 [43 – 61] | 62.5 [54 – 71] | 54.4 [45 – 64] | 11.5 [ 3 – 20] | 47.2 [ 34 – 61] | 9.4 [1.5– 17.3] |
| Sensitivity (%) [95% CI] | 78.9 [75 – 82.7] | 91.1 [ 85.9 – 96.3] | 72.7 [64.5 – 80.8] | 20.0 [9.2 – 30.8] | 14.3 [4.9 – 23.7] | 9.1 [1.4 – 16.8] |
| False negative error rate (%) [95% CI] | 21.1 [13.6 – 28.6] | 8.9 [ 3.7 – 14.1] | 27.8 [19.6 – 36] | 80.0 [69 – 90.8] | 90.0 [81.9– 98] | 87.5 [79 – 96.4] |
| Specificity (%) {95% CI] | 86.0 [ 79.6– 92.4] | 43.1 [34 – 52.2] | 64.4 [55.5– 73.2] | 87.8 [78.6 – 96.6] | 49.6 [36.1 – 63.1] | 89.6 [81.4 – 97.8] |
| False positive error rate (%) [95% CI] | 14.0 [20.4 – 76.6] | 56.9 [48.1– 65.7] | 35.6 [26.8 – 44.4] | 12.2 [3.4– 21] | 50.4 [36.9 – 63.9] | 10.4 [2.2 – 18.6] |
| Positive predictive value (%) [95% CI] | 84.9 [78.3 – 91.5] | 60.7 [51.7 – 69.7] | 65.6 [56.9 – 74.3] | 14.3 [ 9.4– 23.7] | 2.0 [1.0 – 5.8] | 9.1 [1.4– 15.8] |
| Negative predictive value (%) [95% CI] | 80.3 [73 – 87.6] | 83.3 [ 76.5 –90.1] | 71.7 [ 63.4 – 80] | 91.3 [ 87.4– 95.2] | 62.7 [49.7 – 75.7] | 93.5 [86.9 – 100] |
| Diagnostic odds ratio (DOR)^*^ | 23 | 7.7 | 4.8 | 1.8 | 0.1 | 1.1 |

^*^DOR = Diagnostic odds ratio
