# Supplementary material for: Endemic status of urogenital schistosomiasis and the efficacy of a single-dose praziquantel treatment in unmapped rural farming communities in Oyo East Local Government Area, Oyo State, Nigeria
Source: PLoS Negl Trop Dis. 2024 Apr 15;18(4):e0012101. doi: 10.1371/journal.pntd.0012101 (PMC11045121; doi:10.1371/journal.pntd.0012101)
Supplement: S3 Table — (DOCX) [file pntd.0012101.s004.docx]

**S3 Table . Urinogenital schistosomiasis project in Ajagba and Awosan communities in Oyo East Local Government Area**

**field data**

**AJAGBA COMMUNITY**

| Sample/No. | Sex | Age  (yrs) | Haematuria | Eggs/  10 ml urine | Leucocytes | Nitrite | Urobilinogen | Protein | pH | Micro  Haematuria | Specific  Gravity | Ketones | Bilirubin | Glucose |  |
| --- | --- | --- | --- | --- | --- | --- | --- | --- | --- | --- | --- | --- | --- | --- | --- |
| 001 | F | 6 | - | - | - | - | 0.1 | Trace | 6 | - | 1.030 | - | - | - |  |
| 002 | F | 6 | - | >500 | ++125 | Trace | 0.1 | +30(0.3) | 6.5 | +25 | 1.030 | ±5 (0.5) | - | ±100 (5.5) |  |
| 003 | F | 5 | - | 09 | - | Trace | 0.1 | +++300(0.3) | 8.5 | Trace | 1.020 | - | + | ±100(5.5) |  |
| 004 | M | 7 | - | 251 | Trace | - | 0.1 | +++300(0.3) | 6.5 | +++200 | 1.030 | - | - | ±100(5.5) |  |
| 005 | F | 4 | - | - | +70 | - | 0.1 | - | 5 | - | 1.030 | ±5(0.5) | - | - |  |
| 006 | F | 3 | - | - | - | - | 0.1 | - | 6 | Trace | 1.025 | - | - | - |  |
| 007 | M | 3 | - | 55 | - | - | 0.1 | Trace | 6 | +25 | 1.020 | - | - | - |  |
| 008 | M | 8 | - | 18 | - | - | 0.1 | Trace | 6.5 | +++200 | 1.030 | - | - | ±100(5.5) |  |
| 009 | F | 9 | + | >500 | ++125 | - | 0.1 | +30(0.3) | 8 | +++200 | 1.020 | ±5(0.5) | - | - |  |
| 010 | F | 8 | - | 214 | +70 | + | 0.1 | +100(1.0) | 7 | +25 | 1.025 | ±5(0.5) | - | +100(5.5) |  |
| 011 | F | 3 | - | - | +70 | - | 0.1 | Trace | 6 | - | 1.030 | ±5(0.5) | + | - |  |
| 012 | M | 3 | - | - | - | - | 0.1 | - | 5 | - | 1.030 | - | - | - |  |
| 013 | F | 13 | - | 50 | Trace | - | - | Trace | 8 | - | 1.015 | - | - | - |  |
| 014 | F | 8 | - | 28 | +70 | - | 0.1 | ++++1000(10) | 8.5 | +25 | 1.000 | ±5(0.5) | - | - |  |
| 015 | F | 10 | - | 60 | +++500 | - | 0.1 | +30(0.3) | 8.5 | ++80 | 1.015 | - | + | ±100(5.5) |  |
| 016 | F | 6 | - | 12 | +70 | - | 0.1 | +30(0.3) | 8.5 | - | 1.005 | - | - | ±100(5.5) |  |
| 017 | M | 5 | - | 04 | - | - | 0.1 | - | 7.5 | ++80 | 1.020 | - | - | - |  |
| 018 | M | 7 | - | 10 | ++125 | - | 0.1 | trace | 8 | ++80 | 1.015 | - | - | - |  |
| 019 | M | 11 | - | - | - | - | 0.1 | Trace | 7 | - | 1.025 | +5(0.5) | + | ±100(5.5) |  |
| 020 | F | 10 | - | 16 | +70 | - | 0.1 | Trace | 5 | ++80 | 1.025 | - | + | +250(14) |  |
| 021 | F | 4 | - | - | Trace | - | 0.1 | +3 | 8 | - | 1.010 | - | ++ | = |  |
| 022 | F | 7 | - | 302 | ++  +++500 | + | - | ++100(1.0) | 6 | ++80 | 1.030 | ±5(0.5) | + | ±100(5.5) |  |
| 023 | M | 7 | - | 07 | +70 | - | 0.1 | Trace | 6 | - | 1.030 | - | - | - |  |
| 024 | M | 12 | + | 118 | ++125 | - | 0.1 | Trace | 6.5 | +++200 | 1.025 | ±5(0.5) | + | ±100(5.5) |  |
| 025 | M | 40 | - | 01 | Trace | - | 0.1 | - | 7 | - | 1.030 | - | - | = |  |

| 026 | F | 65 | - | - | - | - | - | +30(3.0) | 7.5 | - | 1.005 | - | - | - |  |
| --- | --- | --- | --- | --- | --- | --- | --- | --- | --- | --- | --- | --- | --- | --- | --- |
| 027 | M | 5 | - | - | - | - | 0.1 | - | 8.5 | - | 1.010 | - | - | - |  |
| 028 | F | 2 | - | - | - | - | 0.1 | Trace | 7.5 | - | 1.020 | - | - | - |  |
| 029 | F | 11 | - | >500 | ++125 | - | 0.1 | +++300(3.0) | 8.5 | +25 | 1.010 | - | - | - |  |
| 030 | F | 38 | - | - | - | - | - | - | 6.5 | - | 1.030 | - | = | - |  |
| 031 | F | 10 | - | 03 | ++125 | + | 0.1 | trace | 7.5 | +25 | 1.020 | - | = | - |  |
| 032 | M | 2 | Nd | Nd | Nd | Nd | Nd | Nd | Nd | Nd | Nd | Nd | Nd | Nd | * |
| 033 | F | 35 | - | - | - | - | 0.1 | Trace | 5 | - | 1.030 | ±5(0.5) | - | ±100(5.5) |  |
| 034 | M | 2 | + | 90 | Trace | Trace | 0.1 | ++++1000 (10) | 6.5 | ++80 | 1.025 | ±5(0.5) | + | +250(14) |  |
| 035 | F | 8 | - | 175 | +70 | - | 0.1 | Trace | 6.5 | +++200 | 1.030 | - | - | - |  |
| 036 | F | 27 | - | 02 | +70 | - | 0.1 | Trace | 6.5 | trace | 1.025 | - | - | - |  |
| 037 | F | 6 | - | - | Trace | - | 0.1 | Trace | 5 | +25 | 1.030 | ±5(0.5) | - | - |  |
| 038 | F | 6 | - | - | - | - | 0.1 | Trace | 5 | trace | 1.030 | ±5(0.5) | - | - |  |
| 039 | F | 10 | + | >500 | ++125 | - | 0.1 | ++100(1.0) | 8.5 | +++200 | 1.005 | - | - | - |  |
| 040 | F | 12 | - | 10 | +70 | - | 0.1 | trace | 6 | +++200 | 1.025 | - | - | - |  |
| 041 | F | 13 | - | 05 | +125 | Trace | 0.1 | Trace | 5 | +25 | 1.025 | - | - | - |  |
| 042 | F | 50 | - | - | +70 | - | 0.1 | - | 6 | - | 1.025 | - | - | - |  |
| 043 | F | 30 | - | - | +70 | - | 0.1 | Trace | 6 | - | 1.030 | - | - | - |  |
| 044* | M | 14 | + | >500 | ++125 | - | 0.1 | +30(0.3) | 6.5 | +++200 | 1.030 | - | - | - |  |
| 045 | M | 16 | - | 181 | trace | - | 1 | +++300(30) | 5 | +++200 | 1.030 | +5(0.5) | + | ±100(5.5) |  |
| 046 | F | 53 | - | - | Trace | Trace | 0.1 | Trace | 5 | - | 1.030 | - | - | - |  |
| 047 | M | 53 | - | - | - | - | 0.1 | Trace | 5 | - | 1.030 | - | - | - |  |
| 048 | M | 82 | - | - | - | - | 0.1 | Trace | 6.5 | - | 1.025 | ±5(0.5) | ++ | ±100 |  |
| 049 | F | 48 | - | - | - | - | 0.1 | Trace | 7 | +10 | 1.010 | - | - | - |  |
| 050 | F | 9 | - | 179 | ++125 | - | 0.1 | trace | 8.5 | +25 | 1.010 | - | - | - |  |
| 051 | F | 12 | - | 17 | - | - | 0.1 | +30(0.3) | 8.5 | +25 | 1.010 | - | - | - |  |
| 052 | M | 13 | + | >500 | Trace | - | 0.1 | +++300(3.0) | 6.5 | +++200 | 1.025 | ±5(0.5) | - | - |  |
| 053 | M | 19 | - | 100 | - | - | 0.1 | +30(0.3) | 7.5 | +++200 | 1.010 | - | - | - |  |
| 054 | F | 17 | - | 04 | +70 | - | 0.1 | - | 6 | +25 | 1.030 | - | - | - |  |
| 055 | F | 67 | - | - | ++125 | - | 0.1 | - | 8.5 | - | 1.030 | - | - | - |  |
| 056 | F | 27 | - | - | - | - | 0.1 | - | 6 | - | 1.010 | - | - | - |  |
| 057 | F | 35 | - | - | +70 | - | 0.1 | - | 6 | - | 1.030 | - | - | - |  |
| 058 | F | 64 | - | - | ++125 | + | 0.1 | - | 6 | - | 1.030 | - | - | - |  |
| 059 | F | 68 | - | - | - | - | 0.1 | - | 6 | - | 1.030 | - | - | - |  |
| 060 | F | 46 | - | - | - | - | 0.1 | - | 6 | +10 | 1.030 | - | - | - |  |
| 061 | F | 27 | - | - | +70 | Trace | 0.1 | Trace | 8.5 | - | 1.000 | - | - | - |  |
| 062 | M | 50 | - | 09 | - | - | 0.1 | trace | 8 | Trace | 1.030 | - | - | - |  |
| 063 | M | 61 | - | - | - | - | 0.1 | Trace | 6 | - | 1.030 | - | - | - |  |
| 064 | M | 6 | + | 447 | +70 | - | 0.1 | Trace | 8.5 | Trace | 1.000 | - | - | - |  |
| 065 | F | 4 | - | - | - | Trace | - | Trace | 7 | Trace | 1.015 | - | + | ±100(0.5) |  |
| 066 | M | 8 | + | >500 | ++125 | - | 1(16) | ++++1000(10) | 8.5 | +25 | 1.000 | ±5(0.5) | - | - |  |
| 067 | F | 13 | + | >500 | +70 | - | 0.1 | ++100(1.0) | 8 | +25 | 1.005 | - | + | ±100(0.5) |  |
| 068 | F | 30 | - | 04 | - | - | 0.1 | Trace | 8 | - | 1.010 | ±5(0.5) | - | - |  |
| 069 | M | 29 | - | 02 | ++125 | - | 0.1 | Trace | 6.5 | ++80 | 1.025 | - | - | ±100(5.5) |  |
| 070 | F | 38 | - | - | - | Trace | - | trace | 8 | - | 1.015 | - | - | - |  |
| 071 | F | 58 | - | - | - | - | - | Trace | 6.5 | - | 1.015 | ±5(0.5) | - | - |  |
| 072 | M | 13 | - | 01 | trace | trace | 0.1 | trace | 8 | +25 | 1.025 | - | - | - |  |
| 073 | F | 40 | - | - | +70 | - | 0.1 | Trace | 6.5 | +++200 | 1.030 | ±5(0.5) | - | ±100(5.5) |  |
| 074 | F | 56 | - | 10 | ++125 | Trace | - | ++100(1.0) | 6.5 | Trace | 1.025 | ±5(0.5) | - | ±100(5.5) |  |
| 075 | F | 60 | - | - | Trace | - | - | - | 6 | +25 | - | - | + | - |  |
| 076 | M | 40 | - | - | - | - | - | Trace | 6 | - | 1.030 | ±5(0.5) | - | +250(14) |  |
| 077 | M | 50 | - | - | +70 | - | 0.1 | +30(0.3) | 7.5 | - | 1.015 | - | - | - |  |
| 078 | F | 64 | - | - | - | Trace | 0.1 | Trace | 5 | - | 1,030 | - | - | - |  |
| 079 | F | 12 | - | - | +70 | - | 0.1 | Trace | 6 | - | 1.030 | - | - | - |  |
| 080 | F | 7 | - | - | trace | - | 0.1 | ++100(1.0) | 7 | - | 1.020 | - | - | - |  |
| 081 | M | 14 | - | 31 | ++25 | + | 0.1 | ++100(1,0) | 6 | ++80 | 1.030 | ±5(0.5) | + | ±250(14) |  |
| 082 | M | 43 | - | 01 | +70 | - | 0.1 | Trace | 5 | - | 1.030 | ±5(0.5) | + | ±100(5.5) |  |
| 083 | F | 9 | + | 42 | ++125 | - | 0.1 | ++100(1.0) | 7.5 | +25 | 1.010 | - | - | - |  |
| 084 | F | 8 | - | - | - | - | 0.1 | +30(0.3) | 8 | - | 1.015 | - | - | - |  |
| 085 | M | 5 | - | 04 | +++500 | - | - | Traces | 6 | - | 1.030 | +++80(8) | + | ++500(28) |  |
| 086* | M | 3 | Nd | Nd | Traces | - | 0.1 | +++1000(10) | 8.5 | Trace | 1.000 | ±5(0.5) | - | - | * |
| 087 | F | 31 | - | - | - | - | 0.1 | - | 6.5 | - | 1.020 | - | - | - |  |
| 088 | M | 40 | - | 07 | Traces | - | 0.1 | - | 6 | Trace | 1.005 | - | - | - |  |
| 089 | F | 10 | - | 06 | +70 | - | 0.1 | +++300(3.0) | 8.5 | - | 1.005 | - | - | - |  |
| 090 | F | 50 | - | - | +70 | - | 0.1 | Trace | 6.5 | +25 | 1.000 | - | - | - |  |
| 091 | F | 3 | Nd | Nd | Nd | - | Nd | Nd | Nd | Nd | Nd | Nd | Nd | Nd | * |
| 092 | F | 20 | - | - | traces | - | 0.1 | trace | 6 | trace | 1.000 | - | - | - |  |
| 093 | F | 55 | - | - | - | - | 0.1 | Trace | 6 | - | 1.030 | - | + | - |  |
| 094 | F | 15 | - | 70 | +70 | - | 0.1 | Trace | 6.5 | ++80 | 1.020 | - | - | - |  |
| 095 | F | 12 | - | 14 | - | traces | 0.1 | Trace | 6 | - | 1.030 | +5(0.5) | - | - |  |
| 096 | F | 30 | - | - | - | - | 0.1 | Trace | 8.5 | - | 1.020 | - | - | - |  |
| 097 | F | 45 | - | 06 | - | - | 0.1 | +30(0.3) | 7 | ++80 | 1.010 | - | - | - |  |
| 098 | M | 40 | - | - | - | - | 0.1 | Trace | 6 | - | 1.025 | - | - | - |  |
| 099 | Nd | Nd | Nd | Nd | Nd | Nd | Nd | Nd | Nd | Nd | Nd | Nd | Nd | Nd | * |
| 100 | F | 54 |  | 01 | - | - | 0.1 | Trace | 6.5 | - | 1.030 | - | - | ±100(5.5) |  |
| 101 | M | 13 | - | - | - | - | 0.1 | ++100(1.0) | 6 | - | 1.025 | - | - | - |  |
| 102 | M | 34 | Nd | Nd | Nd | Nd | Nd | Nd | Nd | Nd | Nd | Nd | Nd | Nd | * |
| 103 | M | 74 | - | - | - | - | 0.1 | Trace | 7 | - | 1.010 | - | - | - |  |
| 104 | M | 70 | - | - | - | - | 0.1 | Trace | 6 | +++200 | 1.025 | ±5(0.5) | - | - |  |
| 105 | F | 47 | - | - | - | - | 0.1 | ++100(1.0) | 7.5 | +++200 | 1-010 | - | - | - |  |
| 106 | M | 14 |  | 15 | - | - | 0.1 | Trace | 6 | +++200 | 1.030 | - | - | - |  |
| 107 | M | 14 |  | 58 | - | - | 0.1 | Trace | 7.5 | ++80 | 1.015 | - | - | - |  |
| 108 | M | 15 |  | 40 | - | - | 0.1 | - | 6 | +++200 | 1.030 | - | - | - |  |
| 109 | M | 15 |  | 125 | - | Trace | 0.1 | ++100(0.1) | 8.5 | +++200 | 1.015 | - | - | - |  |
| 110 | M | 50 | - | - | - | - | 0.1 | - | 6 | - | 1,015 | - | - | - |  |
| 111 | F | 39 | - | - | +++125 | - | 0.1 | - | 6 | - | 1.020 | - | - | - |  |
| 112 | M | 12 | - | - | - | - | 0.1 | - | 6 | - | 1.025 | - | - | - | * |
| 113 | M | 8 | - | - | - | - | 0.1 | - | 8.5 | - | 1.050 | - | - | - |  |
| 114 | M | 65 | - | - | Trace | - | 0.1 | - | 8 | - | 1.015 | - | - | - |  |
| 115 | F | 24 | - | - | +70 | - | 0.1 | - | 6 | - | 1.020 | - | - | - |  |
| 116 | F | 2 | - | - | - | - | 0.1 | - | 6 | - | 1.030 | - | - | - |  |
| 117 | M | 40 | - | - | - | - | 0.1 | - | 6 | - | 1.030 | - | - | - |  |
| 118 | M | 43 | - | - | - | - | 0.1 | - | 6 | - | 1.020 | - | - | - |  |
| 119 | M | 6 | - | - | - | - | 0.1 | - | 6 | - | 1.025 | - | - | - |  |
| 120 | M | 4 | - | - | - | - | 0.1 | - | 5 | - | 1.030 | - | - | - |  |
|  |  |  |  |  |  |  |  |  |  |  |  |  |  |  |  |
|  |  |  |  |  |  |  |  |  |  |  |  |  |  |  |  |
|  |  |  |  |  |  |  |  |  |  |  |  |  |  |  |  |
|  |  |  |  |  |  |  |  |  |  |  |  |  |  |  |  |

**AWOSAN COMMUNITY**

| S/No. | Sex | Age  (yrs) | Haematuria | Eggs/  10 ml urine | Leucocytes | Nitrite | Urobilinogen | Protein | pH | Micro  Haematuria | Specific  Gravity | Ketones | Bilirubin | Glucose |  |
| --- | --- | --- | --- | --- | --- | --- | --- | --- | --- | --- | --- | --- | --- | --- | --- |
| 001 | M | 4 | - | - | - | - | 0.1 | - | 6 | - | 1.030 | - | - | - |  |
| 002 | F | 7 | - | - | - | Trace | 0.1 | ++++1000 | 8.5 | - | 1.005 | - | - | - |  |
| 003 | F | 8 | - | - | - | Trace | 0.1 | ++++1000 | 8.5 | - | 1.005 | - | - | - |  |
| 004 | M | 7 | - | - | - | Trace | 0.1 | +++300 | 8 | - | 1.020 | - | - | ±100(5.5) |  |
| 005 | M | 7 | - | - | - | - | 0.1 | ++++1000 | 8.5 | - | 1.010 | - | - | - |  |
| 006 | F | 35 | - | - | - | - | 0.1 | Trace | 8 | - | 1.005 | - | - | - |  |
| 007 | M | 7 | - | - | - | - | 0.1 | +++300 | 8 | - | 1.005 | - | - | - |  |
| 008 | F | 60 | - | - | - | - | 0.1 | - | 5 | trace | 1.020 | ±5(0.5) | - | - |  |
| 009 | F | 10 | - | - | - | - | 1(16) | - | 5 | - | 1.025 | - | - | - |  |
| 010 | M | 10 | - | - | - | trace | 0.1 | Trace | 7.5 | - | 1.025 | - | - | - |  |
| 011 | F | 5 | - | - | - | - | 0.1 | Trace | 8 | - | 1.005 | - | - | - |  |
| 012 | M | 7 | - | - | - | - | 0.1 | +30(0.3) | 8.5 | - | 1.010 | - | - | - |  |
| 013 | M | 11 | - | - | -- | - | 0.1 | +++300(3.0) | 7.5 | Trace | 1.015 | + | - | ±100(5.5) |  |
| 014 | F | 10 | - | - | - | - | 0.1 | +++300(3.0) | 8 | - | 1.-010 | - | - | - |  |
| 015 | F | 7 |  |  | - | - | 0.1 | - | 6 | Trace | 1.025 | + | - | ±100(5.5) |  |
| 016 | F | 7 | - | - | Nd | Nd | Nd | Nd | Nd | Nd | Nd | Nd | Nd | Nd |  |
| 017 | M | 5 | - | - | - | - | 0.1 | Trace | 6 | - | 1.025 | - | = | - |  |
| 018 | M | 6 | - | - | - | - | 0.1 | Trace | 6 | Trace | 1.020 | - | = | ±100(5.5) |  |
| 019 | F | 10 | - | - | - | trace | 0.1 | Trace | 8.5 | - | 1.005 | - | = | - |  |
| 020 | M | 13 | - | - | - | - | 0.1 | ++100(1.0) | 7.5 | - | 1.015 | - | = | - |  |
| 021 | M | 9 | - | - | - | - | 0.1 | +++300(3.0) | 7.5 | Trace | 1.020 | - | - | - |  |
| 022 | F | 4 | - | - | - | - | 0.1 | - | 5 | - | 1.025 | - | - | - |  |
| 023 | M | 4 | - | - | Nd | Nd | Nd | Nd | Nd | Nd | Nd | Nd | Nd | Nd |  |
| 024 | F | 13 | - | - | - | - | 0.1 | Trace | 8 | - | 1.015 | - | - | - |  |
| 025 | F | 10 | - | - | - | - | 0.1 | Trace | 8.5 | - | 1.010 | - | - | - |  |

| 026 | M | 2 | - | - | - | - | 0.1 | - | 5 | - | 1.030 | - | - | - |  |
| --- | --- | --- | --- | --- | --- | --- | --- | --- | --- | --- | --- | --- | --- | --- | --- |
| 027 | F | 60 | - | - | - | - | 0.1 | - | 5 | - | 1.010 | - | - | - |  |
| 028 | F | 18 | - | - | +70 | - | 0.1 | Trace | 5 | +++200 | 1.030 | - | - | - |  |
| 029 | F | 55 | - | - | - | - | 0.1 | - | 5 | - | 1.010 | - | - | - |  |
| 030 | F | 70 | - | - | +70 | - | 0.1 | - | 6 | - | 1.010 | - | - | - |  |
| 031 | F | 45 | - | - | - | - | 0.1 | - | 5 | - | 1.030 | - | - | - |  |
| 032 | F | 11 | - | - | - | - | 0.1 | - | 5 | - | 1.030 | +5(0.5) | - | - |  |
| 033 | F | 7 | - | - | - | - | 0.1 | - | 6 | - | 1.030 | +5(0.5) | - | - |  |
| 034 | F | 30 | - | - | ++125 | + | 0.1 | - | 6 | - | 1.010 | - | - | - |  |
| 035 | F | 33 | - | - | - | - | 0.1 | - | 8 | - | 1.015 | - | - | - |  |
| 036 | M | 35 | - | - | - | - | 0.1 | trace | 6 | - | 1.020 | - | - | - |  |
| 037 | F | 30 | - | - | - | - | 0.1 | trace | 6 | - | 1.020 | - | - | - |  |
| 038 | F | 65 | - | - | - | - | 0.1 | - | 5 | - | 1.020 | - | - | - |  |
| 039 | M | 11 | - | - | - | - | 0.1 | trace | 5 | - | 1-020 | - | - | - |  |
| 040 | M | 20 | - | 28 | - | - | 0.1 | - | 7.5 | - | 1.020 | - | - | - |  |
| 041 | F | 35 | - | 41 | - | - | 0.1 | - | 6 | - | 1.020 | - | - | - |  |
| 042 | F | 38 | - | - | ++125 | - | 0.1 | - | 6 | - | 1.030 | - | - | - |  |
| 043 | F | 19 | - | - | - | - | 0.1 | - | 6 | - | 1.030 | - | - | - |  |
| 044 | M | 2 | - | - | - | - | 0.1 | - | 6 | - | 1.030 | - | - | ±250(1.4) |  |
| 045 | M | 6 | - | - | - | - | 0.1 | trace | 5 | - | 1.030 | - | + | - |  |
| 046 | M | 19 | - | - | - | - | 0.1 | - | 6 | - | 1.030 | - | + | +100(5.5) |  |
| 047 | F | 9 | - | - | - | - | 0.1 | - | 8.5 | - | 1.015 | - | - | - |  |
| 048 | F | 17 | - | - | - | - | 0.1 | - | 6 | - | 1.030 | - | - | - |  |
| 049 | F | 18 | - | 06 | - | - | 0.1 | - | 7.5 | - | 1.025 | - | - | - |  |
| 050 | M | 25 | - | - | - | - | 0.1 | trace | 6 | - | 1.030 | - | - | - |  |
| 051 | F | 9 | - | - | - | - | 0.1 | - | 8 | - | 1.020 | - | - | - |  |
| 052 | F | 50 | - | - | +70 | trace | 0.1 | trace | 7.5 | - | 1.015 | - | - | - |  |
| 053 | M | 60 | - | - | - | - | 0.1 | ++100(1.0) | 8.5 | - | 1.015 | - | - | - |  |
|  |  |  |  |  |  |  |  |  |  |  |  |  |  |  |  |
|  |  |  |  |  |  |  |  |  |  |  |  |  |  |  |  |

- = negative

+ = positive

Nd = not done
